# A Comparative Analysis of Oral Health on the Santo Domingo Pueblo Reservation

**DOI:** 10.1007/s10900-015-0127-9

**Published:** 2015-11-26

**Authors:** Terrence Batliner, Anne Wilson, Elaine Davis, Joaquin Gallegos, Jacob Thomas, Tamanna Tiwari, Karen Fehringer, Katherine Wilson, Judith Albino

**Affiliations:** Centers for American Indian and Alaska Native Health, Colorado School of Public Health, University of Colorado, Anschutz Medical Campus, 13055 E 17th Avenue, F800, Aurora, CO 80045 USA; School of Dental Medicine, Children’s Hospital Colorado, University of Colorado, Anschutz Medical Campus, Aurora, CO USA; Bates College, 2 Andrews Road, Lewiston, ME 04240 USA

**Keywords:** Health care disparities, Oral health, American Indian, Dental decay, Dental sealants

## Abstract

The study was done to compare oral health data from a tribe in a relatively accessible location between Santa Fe and Albuquerque, New Mexico to national American Indian data and broader US data sets. Participants (N = 399) were recruited via random sampling of housing units. Dental health measures included DMFT/dmft and dental sealants. Comparisons were made using data from large-scale oral health surveillance studies. There was no difference in oral health for 3–5 year olds compared to a recent study of AI/AN preschool children. Compared to the general US population, Santo Domingo Pueblo children and adults showed higher prevalence of untreated decay. Children ages 5–19 had higher rates of sealant retention on permanent teeth, and adults showed lower prevalence of complete tooth retention. The children ages 5–19 and 12–19 with at least one sealant have significantly lower DMFT and less untreated decay than those without sealants. However, the percentage of children with and without sealants who had untreated decay was still more than two times higher than the general US population. Oral health of American Indian children and adults in Santo Domingo Pueblo was worse compared to the general US population but similar to previous results reported for the same Indian Health Service Area even though their location is less isolated than many other tribes.

## Introduction

American Indian and Alaska Native (AI/AN) children experience both general [7] and oral health disparities [10] relative to the overall US population. Recent Indian Health Service (IHS) data collected from a community-based sample of 1–5 year old AI/AN children indicated overall decay experience to be 54 % [10], and a study of 3–5 year old AI children residing on the Navajo Nation reported 89 % with decay experience and 72 % with untreated decay [3]. By comparison, National Health and Nutritional Examination Survey data (NHANES 1999–2004) for 2–5 year old children reported decay experience to be 28 % across all ethnic groups (Mexican-American, non-Hispanic Black, and non-Hispanic White) [4]. The severity of dental decay in the primary dentition of AI/AN children further exacerbates the high burden of disease reflected in these prevalence data [1].

AI/AN adults also have poor oral health compared to the general US population. Although few studies have focused on adult populations, a 2010 study using a convenience sample of 135 adult inhabitants of (aged 18 years and older) of the Pine Ridge Reservation in South Dakota found 59 % with moderate to urgent dental care needs [2]. A 1991 study of oral health status, treatment needs, and dental care utilization patterns of a random sample of AI elders (aged 65–74 years) found 58 % to be completely edentulous [9, 16]. The 1991 study also reported high levels of untreated decay among dentate participants (58 %) and low overall dental service utilization; nearly 40 % had not visited a dentist within the last 5 years.

The IHS is composed of 12 administrative service Areas across the country. The IHS has not typically reported oral health data for individual tribes but has produced reports by service Area. Substantial variation in oral health status and untreated decay has been reported across the Areas; [10]. For 2–5 year olds, recent comparisons indicate that preschoolers in the Albuquerque Area, which includes the Santo Domingo Pueblo, have the second highest rate of decay experience and dmft and the third highest level of untreated decay [10].

Two previous studies found high rates of untreated decay in Pine Ridge [2] and Navajo [3]. These studies were conducted in isolated locations with high population to dentist ratios and low average incomes. The study of dental needs on the Santo Domingo Pueblo Reservation was undertaken to examine the oral health of people living in communities with similar economic challenges but less isolation and more focus on prevention through the use of dental sealants. The findings are compared to data from previous studies of AI/AN populations and NHANES.

The Santo Domingo Pueblo is located in Sandoval County, New Mexico, midway between Albuquerque and Santa Fe. It occupies 115 square miles in a high altitude desert environment, and is the fifth largest among the 19 New Mexico pueblos. The 2010 US Census reports the population to be 2456 [15].

Until recently, members of the Santo Domingo Pueblo Tribe received health care from the Santa Fe IHS service unit, which also serves the needs of eight other Pueblo tribes. In an effort to improve health care access and services, Santo Domingo Pueblo Tribal leaders created the Kewa Health Corporation in 2006. In 2012, the Tribe assumed management of all its health services. Its medical facility, the Santo Domingo Health Center, houses the community dental clinic.

## Methods

This study was approved by the Colorado Multiple Institutional Review Board, the Governor of the Pueblo and the CEO of the Kewa Health Corporation of Santo Domingo Pueblo.

Sample size calculations were made to achieve 80 % power to detect differences of 15–20 % in untreated dental decay between the study population and published national survey data [5, 16]. This resulted in a requirement for 348 adult and child participants. The number was rounded up to 400 and a total of 225 homes based on an estimate of two eligible subjects per household. To ensure a representative sample, a random sampling approach was used that had been successfully employed in a previous study with a Native population [13]. Using satellite photographs obtained from tribal authorities, each of the 367 housing structures in the Santo Domingo Pueblo was numbered. Random numbers were then generated, and the first 225 structures selected were visited by one of three research teams. Each research team consisted of a calibrated dental hygienist and a member of the Tribe, designated as the enumerator, who spoke Keresan and was a member of the Community Health Representative program operated by the Tribe. Initial contact with potential study participants was made by the enumerator. The team then visited identified structures/homes to discuss the purpose of the study. Adults were considered eligible if they were between 18 and 83 years of age. Those over the age of 83 were excluded because there were too few to assure their anonymity. Children were eligible if they were between 3 and 17 years of age. If children were not home at the time of the initial visit, the team scheduled a return visit. An effort was made to achieve a balanced number of adult and child participants and no one was included or excluded based on their past use or non-use of dental services.

Each eligible adult was asked to read and sign a consent form, and parents were asked to read and sign consent forms for their children. Children over the age of 7 years were also asked to review an assent form and agree to the dental evaluation. All individuals were given the option to have the consent/assent forms read to them in Keresan. No additional adult participants were recruited once 200 had been surveyed; children continued to be enrolled until a sample of approximately equal numbers of adults and children was obtained. All participants received compensation for their time.

The dental hygienists completed dental evaluations for each consented/assented individual, and the enumerators served as data recorders. The dental evaluation consisted of an intraoral assessment to determine the number of teeth that were decayed, missing, or filled (DMFT/dmft) based on established diagnostic criteria [11, 12]. In addition, the presence of dental sealants was recorded for all children. Prior to data collection, the dental hygienists were calibrated with a gold standard examiner (T. Batliner) for identification and detection of dental caries. Descriptive statistics were generated for prevalence of untreated dental caries, dental restorations (fillings/crowns), dental sealants on permanent teeth, complete tooth retention, and complete tooth loss (edentulism) as well as DMFT/dmft score. Chi-squared tests of independence were used to compare prevalence of untreated dental decay and dental restorations by gender. Consistent with the 2005–2008 NHANES summary report of selected oral health indicators [5], these tests were not age-adjusted. Also consistent with the NHANES report, follow-up gender comparisons were made within each age category. Regression models were used to compare DMFT between children with sealants and those without sealants, controlling for age and gender. Finally, a two-way ANOVA was used to determine differences in mean DMFT/dmft by age and gender.

Results were compared to the 2005–2008 NHANES summary report [5]. NHANES comparisons included prevalence of untreated decay, restorations, sealants on permanent teeth, complete tooth retention, and edentulism. A difference was determined to be statistically significant if the NHANES prevalence value did not fall within the 95 % confidence interval of the corresponding measure in the study data. The NHANES report did not report a sample size, but it is presumed to be large since it was a national survey.

NHANES does not include oral health status for American Indians, so results were also compared to 1999 IHS survey data on AI/AN dental patients residing in the Albuquerque service area [16]. The IHS data are for individuals seeking dental care, but provide the only information by which to compare dental health for American Indian adults and children ages six and older. The 1999 IHS report included children aged 2–5 so a direct comparison could not be made for the 3–5 year olds in this study. Instead, the data for ages 3–5 were compared to a recent national study of AI/AN preschool children [10].

The IHS comparisons included mean DMFT/dmft, using a generalized linear model with a Poisson link function and stratified age as a categorical predictor, and prevalence of untreated dental decay, using a Cochran-Mantel–Haenszel statistic [8] to control for age. Comparisons for 3–5 year olds included prevalence of untreated decay and mean dmft. These comparisons were considered statistically significant if the 95 % confidence intervals did not overlap. All analyses were conducted using the SAS 9.3 package (SAS Institute, Cary, North Carolina). A significance level of .05 was used for all tests.

## Results

Data were collected from 399 participants (195 adults, ages 18–83 years and 204 children ages 3–17 years). Six adult individuals declined participation after the enumerator explained the purpose of the study.

Prevalence data for untreated dental decay and restorations (Tables 1, 2) indicate that oral health status, in general, declined with age, with the highest prevalence of both untreated dental decay (71 %) and dental restorations (91 %) in the 20–64 year old age group. There were no overall gender differences in prevalence of either untreated dental decay (59.7 % females vs 59.6 % males) or dental restorations (79.1 % of females vs 66.5 % of males). However, within the 5–19 year age group, a statistically significant higher prevalence of dental restorations was found for females than for males (76.5 vs 61.9 %, respectively, *p* = 0.042). Table 3 reports mean DMFT/dmft by age and gender. ANOVA results indicated a significant difference in mean DMFT/dmft by age (*p* < 0.0001), with significantly higher mean for adults ages 20–64 than for either 3–4 or 5–19 year old children. Subjects ages 65 and older were excluded from the analysis because of a high rate of complete tooth loss (34.5 %). Mean DMFT/dmft was also significantly higher for females than for males (*p* = 0.01). There was no significant interaction between age and gender (*p* = 0.10).

**Table 1:** Prevalence of untreated dental decay (%) by age and gender (dentate subjects)

Gender	Age in years				Total
	3–4 (n = 50)	5–19 (n = 165)	20–64 (n = 137)	≥65 (n = 19)	
Male (n = 161)	40.7	57.1	75.6	60.0	59.6
Female (n = 210)	52.2	51.9	68.5	42.9	58.6
Total (n = 371)	46.0	54.6	70.8	47.4	59.0

**Table 2:** Prevalence of dental restorations (%) by age and gender (dentate subjects)

Gender	Age in years				Total
	3–4 (n = 50)	5–19^a^ (n = 165)	20–64 (n = 137)	≥65 (n = 19)	
Male (n = 161)	44.4	61.9	86.7	80.0	66.5
Female (n = 210)	34.8	76.5	92.4	78.6	79.1
Total (n = 371)	40.0	69.1	90.5	79.0	73.6

^a^Significant difference by gender (*p* < 0.05)

**Table 3:** DMFT/dmft by age and gender

Age	Gender	N	Mean (SD)	Total
3–4	Female	23	5.2 (4.5)	5.7 (4.8)
	Male	27	6.1 (5.0)	n = 50
5–19	Female	81	5.6 (4.1)	5.1 (4.0)
	Male	84	4.6 (3.8)	n = 165
20–64	Female	95	15.0 (6.9)	14.0 (6.9)
	Male	47	12.1 (6.5)	n = 142

Regression analysis indicated a significant difference in mean DMFT between children with at least one sealant and those children without any sealants (*p* = 0.003), (Table 4). Children with at least one sealant had lower DMFT and lower prevalence of untreated decay.

**Table 4:** Sealants, DMFT and untreated decay

	N	Mean (DMFT)	SD	*p* value for difference	% Untreated Decay
Ages 5–19					
Sealants	87	2.57	2.97	0.003	37.9
No sealants	66	3.62	4.18		43.9
Ages 12–19					
Sealants	45	3.1	3.3	0.003	40.0
No sealants	25	7.2	4.7		68.0

The number and percentage of participants with DMFT = 0 and dmft = 0, in other words those with no decay or history of decay, were examined by age (Table 5). This group declined with age as expected but contrary to other studies [2, 3, 10] more males were in this group than females for the 5–19 age group and the 20–64 age group.

**Table 5:** The “caries free” cohort

Age	Gender	N	% DMFT = 0 and dmft = 0	Total
3–4	Female	23	26.1	24.0
	Male	27	22.2	n = 50
5–19	Female	81	4.9	11.5
	Male	84	17.9	n = 165
20–64	Female	95	1.1	1.4
	Male	47	2.1	n = 142

A number of statistically significant differences were found when results were compared to 2005–2008 NHANES report (Table 6). The prevalence of untreated dental decay was significantly higher in the current study than for NHANES (61.5 vs 21.5 %, respectively), as was the prevalence of sealants on permanent teeth among 5–19 year olds (57 vs 27 %). Complete tooth retention among adults ages 20–64 years was significantly lower among current study participants (33 vs 49 %), and complete tooth loss among adults ages 65 years and older was higher in the current study (34.5 vs 23 %), although this difference was not statistically significant.

**Table 6:** Prevalence of oral health indicators, current study versus 2005–2008 NHANES

Oral health indicator	Current study (%) [95 % CI]	NHANES (%)
Untreated decay^a^	61.5 [56.2, 66.9]	21.5^b^
Restorations^a^	78.8 [74.3, 83.2)]	75.5
Sealants, permanent teeth (ages 5–19)	56.9 [48.9, 64.8]	27.2^b^
Complete tooth retention (ages 20–64)	33.1 [25.4, 40.9]	48.6^b^
Complete tooth loss (ages 65+)	34.5 [16.1, 52.9]	22.9

^a^Ages 3 and 4 excluded (not reported in NHANES study)

^b^Statistically significant difference from current study

Compared to the 1999 IHS user population, the current study population had significantly lower mean DMFT/dmft (F_4, 236_ = 14.86, *p* < 0.0001) when categorized by equivalent age groups. There was no statistically significant difference in prevalence of untreated decay in permanent teeth between the IHS and current study populations after controlling for age (Cochran-Mantel–Haenszel χ^2^ = 2.24, *p* = 0.13).

## Discussion

The study results indicate that American Indians living on the Santo Domingo Pueblo reservation have significantly worse oral health compared to the general US population [5]. However, school-aged children were found to have significantly lower mean DMFT/dmft than earlier cohorts from the same IHS Area (Indian Health Service 2000) but the previous study included only those who sought dental care, and thus may reflect a population with greater overall treatment needs.

No differences in oral health measures were seen for preschool aged children when compared to results from a recent national survey of AI/AN preschool children [10]. In other words, the Santo Domingo preschool children were near the IHS average for the oral health elements measured. The fact that the Santo Domingo Pueblo Tribe is less isolated than other Tribes does not seem to be a factor related to the oral health of children. They are typical of Native children examined across the US which means nearly half have untreated decay, and the percentage of children ages 5–19 with untreated decay is over three times that of the general US population (55 % compared to 17 %) [5].

There were some important unanticipated findings with this study: As seen in Table 4, as the children get older the difference in DMFT between those with sealants and those without sealants increases. The children with sealants at ages 12–19 have significantly lower DMFT and untreated decay than children without sealants. Why this is the case is unknown. One could speculate, that the children who have retained sealants could have parents or care givers who are more aware of oral health and ensure more effective oral health behaviors at home, the sealants could be causing the difference or there could be another reason for the finding. More research is required to identify the reason for the benefit. However, it is still troubling to see the percentage of participants with untreated decay in the groups with and without sealants over two times higher than the general US population. This may be due to insufficient access to restorative care.

The group with no decay in this study is unique (Table 5). As expected, the “caries free” participants with no active decay or decay experience is small in the youngest age groups and declines with age but additional research should be undertaken to identify why more males than females in the two older age groups have no history of decay. Table 5 is consistent with Table 3, which indicates higher DMFT/dmft in female participants. Most previous studies have reported the opposite, with males having higher rates of active decay and greater decay experience than females [6, 14]. Again more research would be needed to determine the cause for this finding.

The strengths of this work are the sampling methodology and the involvement of the community in conducting the study. Using satellite photographs to identify structures on the reservation and then randomly selecting those structures to visit for recruitment ensured a representative sample of people living on the reservation was attained. This could not have been done without the involvement of community members who helped with the study. Their work made it possible to recruit people in their homes and also ensured the research teams performed in ways acceptable to Tribal leaders and the Reservation population.

The first limitation of the study involves the comparison to the 1999 IHS survey results (Indian Health Service 2000). The current study involved a random sample of people living on the reservation. The 1999 IHS study examined users of the dental clinics. Dental clinic users may have more dental needs than a random sample of the community so the comparison of DMFT/dmft between the two studies should be considered cautiously.

Another limitation is the inability to determine why the findings exist. An examination of participant behaviors, knowledge, attitudes and beliefs was not conducted so correlations among these elements and the dental data are not possible.

## Conclusion

Children and adults living on the Santo Domingo Pueblo reservation have significant unmet oral health needs. Substantial efforts to prevent decay through the use of dental sealants do seem to have a positive effect but there is still a high percentage of children and adults with untreated decay. This may indicate a lack of access to restorative care. Efforts should be considered to improve access to both preventive and restorative care. Clearly, in this population and all populations, existing lesions must be restored and new lesions must be prevented in order to substantially improve oral health. Interventions that focus only on prevention or only on restorative treatment are likely to be insufficient to significantly improve oral health.
